# Green's Encyclopædia Medica

**Published:** 1915-12

**Authors:** 


**Green's Encyclopædia Medica.** Second Edition. Vol. II. Aspiration to Chlorodyne. Pp. viii. 677. Edinburgh and London: W. Green and Sons. 1915. Price 20s. net.

Dr. J. M. Ballantyne continues the general editorship of this great work, which is now too well known to need any description or criticism. Amongst the articles revised by their authors we notice that of Dr. P. Watson-Williams on Bronchitis.

